# Proteomic and Immunochemical Characterization of Glutathione Transferase as a New Allergen of the Nematode *Ascaris lumbricoides*

**DOI:** 10.1371/journal.pone.0078353

**Published:** 2013-11-04

**Authors:** Nathalie Acevedo, Jens Mohr, Josefina Zakzuk, Martin Samonig, Peter Briza, Anja Erler, Anna Pomés, Christian G. Huber, Fatima Ferreira, Luis Caraballo

**Affiliations:** 1 Institute for Immunological Research, University of Cartagena, Cartagena, Colombia; 2 Foundation for the Development of Medical and Biological Sciences, Cartagena, Colombia; 3 Department of Molecular Biology, Division of Chemistry and Bioanalytics, University of Salzburg, Salzburg, Austria; 4 Department of Molecular Biology, Division of Allergy and Immunology, University of Salzburg, Salzburg, Austria; 5 Indoor Biotechnologies Inc., Charlottesville, Virginia, United States of America; UNIFESP Federal University of São Paulo, Brazil

## Abstract

Helminth infections and allergy have evolutionary and clinical links. Infection with the nematode *Ascaris lumbricoides* induces IgE against several molecules including invertebrate pan-allergens. These antibodies influence the pathogenesis and diagnosis of allergy; therefore, studying parasitic and non-parasitic allergens is essential to understand both helminth immunity and allergy. Glutathione transferases (GSTs) from cockroach and house dust mites are clinically relevant allergens and comparative studies between them and the GST from *A. lumbricoides* (GSTA) are necessary to evaluate their allergenicity. We sought to analyze the allergenic potential of GSTA in connection with the IgE response to non-parasitic GSTs. IgE to purified GSTs from Ascaris (nGSTA and rGSTA), house dust mites (rDer p 8, nBlo t 8 and rBlo t 8), and cockroach (rBla g 5) was measured by ELISA in subjects from Cartagena, Colombia. Also, multidimensional proteomic approaches were used to study the extract of *A. lumbricoides* and investigate the existence of GST isoforms. We found that among asthmatics, the strength of IgE levels to GSTA was significantly higher than to mite and cockroach GSTs, and there was a strong positive correlation between IgE levels to these molecules.

Specific IgE to GSTA was found in 13.2% of controls and 19.5% of asthmatics. In addition nGSTA induced wheal and flare in skin of sensitized asthmatics indicating that it might be of clinical relevance for some patients. Frequency and IgE levels to GSTA were higher in childhood and declined with age. At least six GST isoforms in *A. lumbricoides* bind human IgE. Four isoforms were the most abundant and several amino acid substitutions were found, mainly on the N-terminal domain. In conclusion, a new allergenic component of Ascaris has been discovered; it could have clinical impact in allergic patients and influence the diagnosis of mite and cockroach allergy in tropical environments.

## Introduction

Allergic diseases such as asthma are public health problems and, together with other immune mediated diseases, are increasing worldwide [1]. In the tropics, helminth infections are also very frequent, for example, *Ascaris lumbricoides* infects around 2 billion people and may influence the pathogenesis, evolution and diagnosis of allergic diseases [2], [3]. Studying the relationships between these conditions has contributed to understanding helminth immunity and allergy. *A. lumbricoides* is very allergenic and there is evidence that it may enhance Th2 responses (reviewed in [4]). Still, its allergens have not been fully identified. There is cross reactivity among several IgE-binding components of Ascaris and other invertebrates such as domestic mites [5]–[7] and cockroaches [8]. Because of their potential impact on protective immunity to Ascaris and the pathogenesis and diagnosis of allergic diseases (e.g. asthma), the characterization of immunogenic and allergenic components of Ascaris is essential. Two allergens from this nematode have been described (Asc s 1 and Asc l 3) and we have evidence that the glutathione transferase of *Ascaris suum* also binds IgE [8]. Therefore, it is important to characterize this potentially allergenic molecule. The glutathione transferases (GSTs) (EC 2.5.1.18) are detoxification enzymes found in most living organisms [9], however, those from invertebrates can induce IgE sensitization in humans and be of clinical relevance for some allergic patients [10]–[13]. The most important known sources of allergenic GSTs are cockroaches, house dust mites and molds (i.e. *Alternaria alternata*). Based on the strength of the specific IgE response and the frequency of sensitization, cockroach GST (Bla g 5) is considered the most allergenic [10], [13], followed by the GST of the house dust mite *Dermatophagoides pteronyssinus* (known as Der p 8). The prevalence of IgE sensitization to GSTs is higher in allergic patients living in tropical environments [12], [14], [15] compared to those from temperate areas [16]–[18]. The factors influencing the allergenicity of invertebrate GSTs are unclear. There is evidence suggesting that they exist as isoforms in cockroach, house dust mites and nematodes [12], [19], [20].

GSTs from helminths also induce IgE antibodies in infected individuals [21]. For example, specific IgE antibodies to *Schistosoma ssp*., GST have been detected in endemic communities [22], [23] but this response has been related to protective immunity rather than allergic symptoms [24]. Given the structural homology between parasitic and non parasitic GSTs it has been hypothesized that co-exposure might affect the frequency of sensitization and the strength of the serological response. Indeed, there is experimental evidence that molecular mimicry between the GSTs from the nematode *Wuchereria bancrofti* and cockroach might boost cross-sensitization [25]. Recent advances in protein analysis have made possible the analysis of minute amounts of single molecules and the study of natural isoforms from complex protein extracts [26]. In this study, we sought to purify the GSTs from *A. lumbricoides* (GSTA) to study their IgE binding properties and allergenic potential. We found that GSTA has allergenic properties. Serum specific IgE levels to this molecule positively correlate with the IgE levels to mite and cockroach GSTs. In addition, there are at least six GSTA isoforms that are recognized by human IgE, one being the most prominent. Furthermore, natural GSTA (nGSTA) induced type-I hypersensitivity reactions in sensitized subjects.

## Results

### Human IgE binds to Glutathione Transferases of *A. lumbricoides*

**Figure 1:**
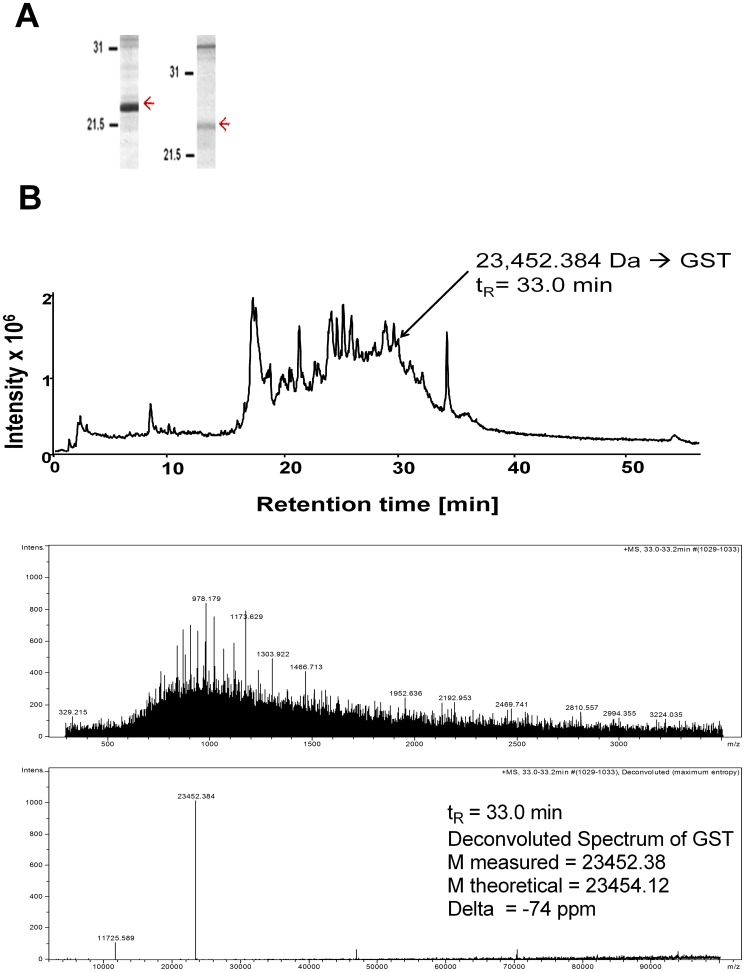
Identification of the 23 kDa component in the Ascaris extract. (A) SDS-PAGE and immunoblotting using pooled sera from asthmatic patients sensitized to Ascaris. The arrows indicate the IgE binding component of ∼23 kDa in the extract. (B) Reconstructed total ion current chromatogram of *A. lumbricoides* extract and mass spectra of the 23 kDa component identified as GST.

**Figure 2:**
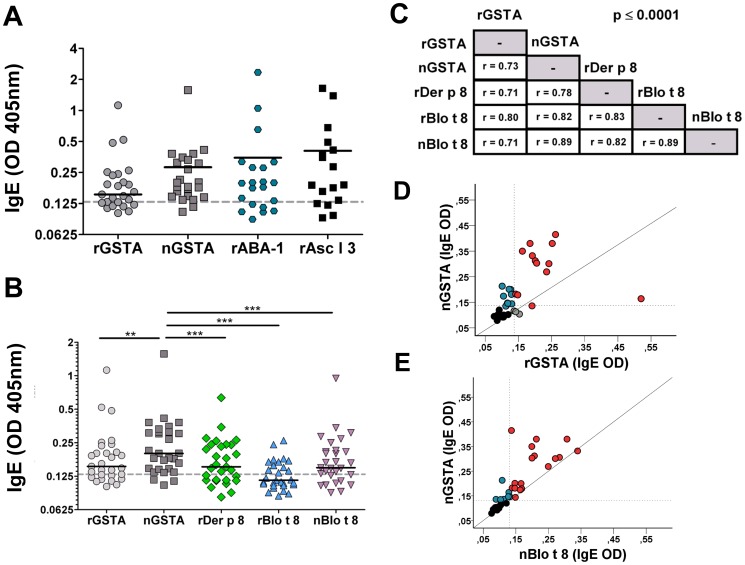
IgE reactivity of human sera to *A. lumbricoides* GST. (A) IgE levels to the natural Ascaris GST (nGSTA) and the recombinant Ascaris GST (rGSTA) in 29 subjects sensitized to the source (26 asthmatics and 3 non-asthmatic controls). The allergens rABA-1 and tropomyosin (rAsc l 3) are presented as positive control for the reactivity of these sera to other Ascaris allergens. (B) IgE levels to purified mite GSTs (rDer p 8, nBlo t 8 and rBlo t 8) in sera from 29 individuals sensitized to Ascaris. Each dot indicates an individual serum. ** p<0.001, *** p<0.0001. The dotted line indicates the cut-off value for positivity to GSTs (OD_405_≥0.13). Solid bar indicate median. (C) Correlation between specific IgE levels to Ascaris and mite GSTs (n = 36); r =  Spearman coefficient. (D) Correlation of specific IgE levels between the nGSTA and the rGSTA. (E) Correlation of specific IgE levels between the nGSTA and nBlo t 8. For figures D and E, red dots indicate individuals with dual-sensitization; blue dots indicate individuals mono-sensitized to nGSTA and black dots non-sensitized subjects.

We previously observed that sera from asthmatic patients sensitized to Ascaris recognized an IgE binding component of approximately 23 kDa in the Ascaris extract [6] (Figure 1A). To further analyze this component, the *A. lumbricoides* extract was separated by Ion-Pair Reverse-Phased micro High Performance Liquid Chromatography (IP-RP-µHPLC) and each fraction was analyzed by mass spectrometry. The component of 23 kDa was detected at 33 minutes of retention time with the mass spectrum of glutathione transferase (Figure 1B); and dot blot screening using pooled human sera revealed a positive IgE binding signal in this HPLC-fraction (data not shown). The human IgE reactivity to Ascaris GST was then tested by ELISA in 29 sera from individuals with positive IgE to the *Ascaris ssp*. extract, including 26 asthmatics and 3 non-asthmatic controls (file S1). Serum IgE antibodies recognized both the nGSTA (Median OD_405_ = 0.200; IQR 0.145–0.312) as well as the recombinant GST1 from *A. suum* (rGSTA, Median OD_405_ = 0.153; IQR 0.124–0.220), but median IgE levels against the nGSTA were significantly higher than those to the rGSTA (Wilcoxon signed rank test, p = 0.004). In addition, serum IgE levels to nGSTA were comparable to the specific IgE levels to other Ascaris allergens such as the nematode-specific rABA-1 (Median OD_405_ = 0.188; IQR 0.112–0.288) and tropomyosin (rAsc l 3, Median OD_405_ = 0.190; IQR 0.130–0.453) (Figure 2A).

Since house dust mites are a source of allergenic GSTs [12], we analyzed the sensitization profile of these sera to purified GSTs from *D. pteronyssinus* (rDer p 8) and *Blomia tropicalis* (rBlo t 8 and nBlo t 8). Interestingly, 20 out of 26 asthmatics sensitized to Ascaris GSTs had also detectable specific IgE to at least one of the mite GSTs (file S1). Wilcoxon matched-pairs signed rank tests showed that median IgE levels to nGSTA were significantly higher than to rBlo t 8 (Median OD_405_ = 0.115; IQR 0.104–0.162, p<0.0001), nBlo t 8 (Median OD_405_ = 0.149; IQR 0.120–0.210, p<0.0001), and rDer p 8 (Median OD_405_ = 0.152; IQR 0.115–0.240, p = 0.0002, Figure 2B). Similar results were obtained when these comparisons were done by parametric paired *t*-tests using log_10_-transformed OD values (data not shown). Specific IgE levels to Ascaris and mite GSTs showed significant positive correlations (Spearman Rank tests, n = 36, Figure 2C). Since the IgE levels to rGSTA were highly correlated with those to nGSTA (r = 0.73, p<0.0001, Figure 2D), the recombinant molecule was found suitable to perform seroprevalence studies in a larger sample set. Among heterologous sources, the strongest correlations were observed between the specific IgE levels to the nGSTA and nBlo t 8, and even though most of the individuals were below the cut-off for positivity or harboring low IgE levels, there was a cluster with clear poly-sensitization to Ascaris and mite GSTs (Figure 2E). To *in vivo* evaluate the allergenic activity of GSTA, the affinity-purified nGSTA and nBlo t 8 were skin tested in eight asthmatic patients and two parasited children with detectable serum IgE antibodies to rGSTA, plus two non-sensitized individuals as controls (Table 1). The nGSTA induced wheal and flare in 5 out of 10 subjects supporting that it is able to trigger mediator release upon IgE binding. Some of them also had type-I hypersensitivity reactions induced by nBlot 8 suggesting that specific IgE sensitization to these molecules might be of relevance for patients poly-sensitized to Ascaris and mite GSTs. In addition, the fact that some patients had hypersensitivity to nBlot 8 but not nGSTA (IDs 284 and 63, Table 1) pointed out that cross-reactive and species-specific epitopes may be involved in the IgE response to GSTs.

**Table 1:** Skin prick tests using purified nGSTA and nBlo t 8.

#	ID	Age	Diagnosis	IgE rGSTA (OD_405nm_)	Glycerol^a^	nGSTA^a^	nBlo t 8^a^	IgE Ascaris(kU/l) b	Ascaris extract^a^	IgE Blomia(kU/l)^b^	Blomiaextract^a^
1	169	10	Asthma	0.240	0 (0)	**3 (10)**	**3 (12)**	0.79	4 (4)	35.0	5 (20)
2	284	56	Asthma	0.261	0 (0)	0 (0)	**3 (20)**	1.65	3 (15)	42.4	7 (30)
3	203	63	Asthma	0.203	1 (1)	**4 (15)**	3 (3)	0.11	3 (15)	3.95	5 (20)
4	125	43	Asthma	0.186	1 (1)	0 (0)	0 (0)	1.83	3 (20)	0.68	1 (1)
5	245	11	Asthma	0.141	1 (1)	**3 (10)**	2 (2)	1.84	4 (10)	90.7	5 (10)
6	151	22	Asthma	0.181	0 (0)	0 (0)	0 (0)	1.59	2 (10)	0.4	0 (0)
7	63	37	Asthma	0.141	0 (0)	0 (0)	**3 (15)**	0.17	5 (15)	1.14	5 (20)
8	310	22	Asthma	n.d.	0 (0)	**4 (4)**	2 (2)	n.d.	3 (3)	n.d.	4 (15)
9	N056	4	Parasited	3.5	0 (0)	**3 (5)**	**3 (5)**	0.01	2.5 (15)	0.7	2 (15)
10	N179	4	Parasited	0.839	0 (0)	1 (2)	2 (2)	4.05	5 (10)	0.91	3 (10)
11	272	36	Asthma	0.093	0 (0)	0 (0)	0 (0)	1.67	4 (15)	2.26	7 (20)
12	002	46	Asthma	0.093	0 (0)	0 (0)	0 (0)	0.03	2(2)	0.02	0 (0)

OD: Optical density units.

a wheal diameter (erythema), mm.

b kU/l as determined by ImmunoCAP®.

**Figure 3:**
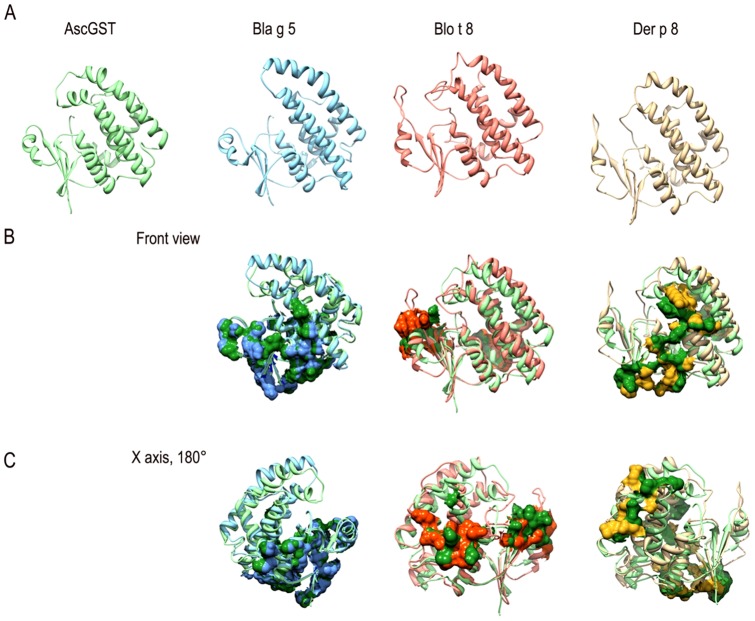
Predicted tertiary structures of Ascaris and non-parasitic GSTs. (A) Ribbon representations of the predicted structures of Ascaris GST1, cockroach GST (Bla g 5) and house dust mite GSTs (Der p 8 and Blo t 8). (B) Superposition of *A. suum* GST1 with Bla g 5, Blo t 8 and Der p 8. Patches of solvent-accessible and conserved residues are shown in surface view. (C) 180° rotation (Y axis) of all structures to visualize other conserved regions.

Multiple alignments of the primary sequences of Ascaris GST1 and other invertebrate GSTs revealed sequence similarities of 50% with Bla g 5, 43% with Blo t 8 and 48% with Der p 8 (file S2). The superposition of the predicted models for the tertiary structure of Ascaris GST1 with Blo t 8, Der p 8 and the cockroach allergen Bla g 5 led to identify several clusters of conserved and surface-exposed residues (Patches) that could represent cross-reactive and species-specific epitopes. Conserved areas varied among the compared allergens and Ascaris GST1 shared more surface areas with Bla g 5 than with the other allergens (Figure 3). The amino acids included in shared patches are shown in file S2.

### Seroprevalence of IgE antibodies to rGSTA and the effect of age

**Figure 4:**
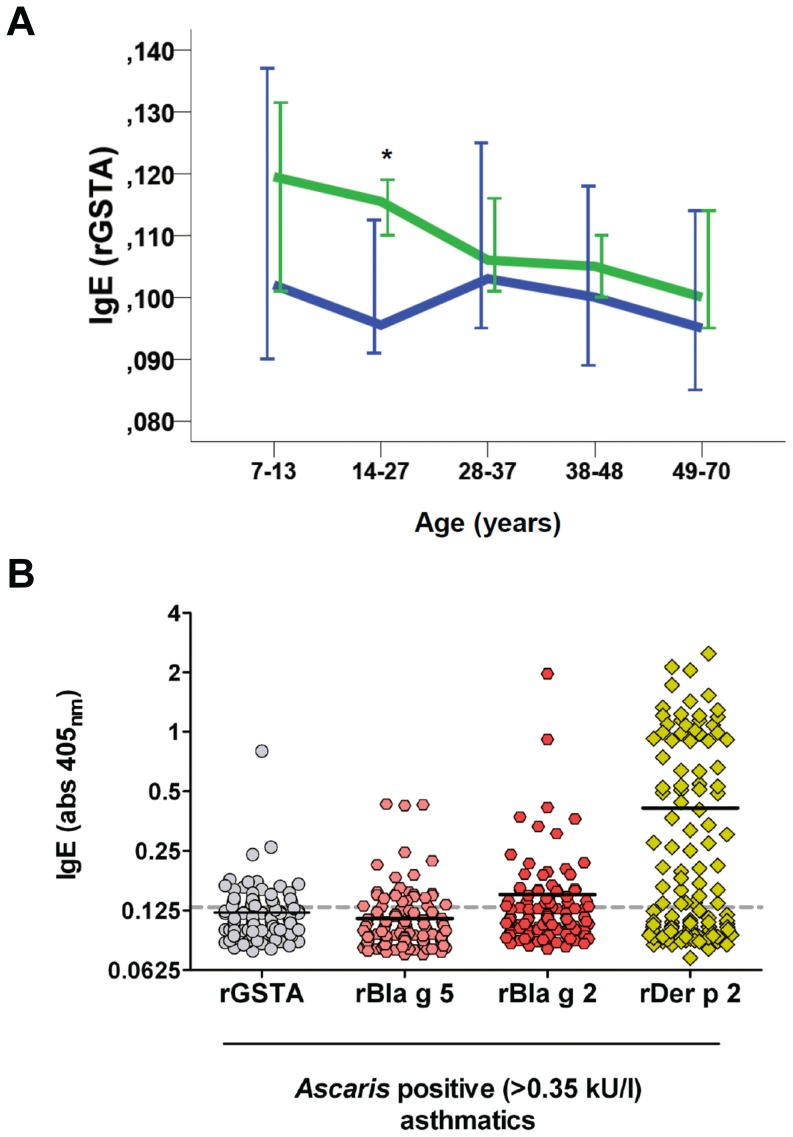
Frequency and strength of the IgE reactivity to rGSTA. (A) Specific IgE levels to rGSTA according to age groups. Green line represents asthmatics and blue line represents non-asthmatic controls. Error bars indicate 95%CI of the median IgE levels (B) A comparison between the strength of specific IgE levels to rGSTA and the cockroach GST (Bla g 5) in asthmatic patients sensitized to Ascaris (ImmunoCAP® >0.35 kU/l, n = 128). IgE levels to rBla g 2 and rDer p 2 were used as reference for the strength of IgE binding to non-GST allergens. Each dot indicates an individual serum.

We investigated the frequency of IgE sensitization to rGSTA and the strength of the response in 306 randomly selected sera from individuals living in Cartagena, Colombia (Mean age 31.8±16.1). There were no differences in the prevalence of positive IgE reactivity to rGSTA between asthmatic patients and non-asthmatic controls (19.5% vs. 13.2% respectively, χ2 = 1.77, p = 0.19). Considering that age have been described as a predictor of the antibody response to parasite GSTs, the study participants were stratified according to age's percentiles in five groups of comparable size. We found that the frequency of positive IgE to rGSTA was significantly greater in children with age 7–13 years (35.2%, n = 62) than to individuals in the groups of 14–27 years (13.6%, n = 66), 28–37 years (14.8%, n = 61), 38–48 years (11.7%, n = 60) or 49–70 years (12.3%, n = 57, χ^2^ = 17.6, df = 4, p = 0.002). Also, age was inversely related to the levels of IgE to rGSTA as a continuous variable (r = −0.18, p = 0.002). There was no difference in the specific IgE levels to rGSTA between cases and controls after adjusting by age using linear regression models (Table 2); and similar results were observed by non-parametric comparisons per age group (Figure 4A). Still, there was a tendency of higher IgE levels to rGSTA in asthmatic patients (Median OD_405_ = 0.115; IQR 0.102–0.124, n = 42) compared to controls (Median OD_405_ = 0.095; IQR 0.091–0.116, n = 24) in the age group of 14–27 years (Mann Whitney *U* test, p = 0.012, 10 000 permutations, Figure 4A). This finding was no longer significant after Bonferroni correction (p = 0.06).

**Table 2:** Descriptive of IgE levels in asthmatics and controls (n = 306).

Variable	Non-asthmatic controls (n = 91)	Asthmatic patients (n = 215)	P value
Age (Mean±SD)	31.5±15.4	31.9±16.4	0.84
Gender (female %)	55 (60.4%)	156 (72.7%)	0.03
Total IgE (kU/l)^a^	91.5 (40.3 – 207.5)	390.3 (148–731)	<0.0001
IgE *D. pteronyssinus* (kU/l)^a^	0.06 (0.03 – 0.16)	1.28 (0.09 – 15.9)	<0.0001
IgE *B. tropicalis* (kU/l)^a^	0.04 (0.01 – 0.14)	2.51 (0.18 – 18.6)	<0.0001
IgE Ascaris (kU/l)^a^	0.15 (0.04 – 0.48)	0.59 (0.17 – 3.10)	<0.0001
IgE+ Ascaris, n (%)^b^	28 (30.8%)	128 (59.5%)	<0.0001
IgE rGSTA (OD)^a^	0.100 (0.091– 0.117)	0.109 (0.095 – 0.124)	0.48^d^
IgE+ rGSTA, n (%)^c^	12 (13.2%)	42 (19.9%)	0.15^d^

OD: Optical density.

a Median (Inter Quartile Range).

b IgE+ Ascaris  =  specific IgE levels to Ascaris >0.35 kU/l.

c IgE+ rGSTA  =  specific IgE levels to rGSTA >0.130 OD units.

d Regression model adjusted by age and gender.

Considering the potential structural homology between Ascaris and cockroach GSTs, and the fact that cockroach GSTs has been described as a potent allergen in sub-tropical environments, we compared the strength of specific IgE levels to rGSTA with that to cockroach GST (rBla g 5) and other non-GST allergens such as Bla g 2 and Der p 2. All the asthmatic patients with a positive IgE sensitization to Ascaris were selected for analysis (n = 128, Mean age 29.9±16.5 years). We found a significant correlation between the IgE levels to rGSTA and rBla g 5 (r = 0.41, p<0.0001) but not between the IgE levels to rGSTA and rDer p 2 (r = 0.11, p = 0.21). Specific IgE levels to rGSTA (Median OD_405_ = 0.113; IQR 0.098–0.126) and to the cockroach rBla g 5 (Median OD_405_ = 0.094; IQR 0.087–0.120) were low with IgE levels to rGSTA being significantly higher than to rBla g 5 (p = 0.0001); however, compared to Der p 2 (Median OD_405_ = 0.130; IQR 0.097–0.541), IgE levels to GSTs were typically of low intensity (Figure 4B). The presence of circulating specific IgE antibodies to Ascaris (Median 2.54 kU/l; IQR 0.79–6.42) and to the cockroach allergen rBla g 2 (Median 0.113; IQR 0.09–0.138) indicated that these patients have been exposed to the allergenic sources and that the observed low IgE reactivity may be a general feature of the response to both parasitic and non-parasitic GSTs.

### GST exists as isoforms in *A. lumbricoides*

**Figure 5:**
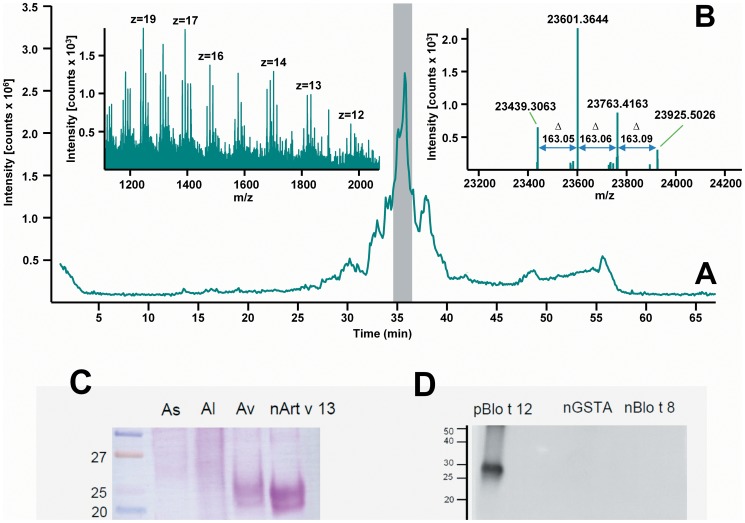
Intact monoisotopic masses of the *A. lumbricoides* GST isoforms. (A) Chromatogram and spectrum of the nGSTA. Injection volume: 15 µl (partial loop); column: 50×1 mm i.d. ProSwift® PS-DVB monolith; eluent A: water +0.05% TFA; eluent B: acetonitrile +0.05% TFA; gradient: 20–50% B in 45 min; flow rate: 60 µl/min; temperature: 60°C. (B) Deconvoluted mass spectrum containing the intact monoisotopic masses of the GST and their relative labeling based on the monoisotopic natural GST. (C) Periodic acid Schiff's staining of the Ascaris extracts in the range of 20–27 kDa. The extract of *Artemisia vulgaris* and the purified natural Art v 13 were used as positive controls of glycosylated allergens. (D) Enhanced chemiluminiscence analysis of glycosylation in the nGSTA and nBlo t 8. The recombinant allergen pBlo t 12.0101 produced in *Pichia pastoris* was used as positive control of glycosylated allergen.

The affinity purified nGSTA was separated by µHPLC and analyzed by Fourier Transform Mass Spectrometry (FT-MS) using a LTQ Linear Ion Trap Orbitrap XL™ mass spectrometer. The corresponding chromatogram included several peaks in the range of 25 to 45 min. The largest peak was found at a retention time of 35.75 min (Figure 5A). Four intact monoisotopic masses were the most abundant in nGSTA (23,439.30 Da, 23,601.36 Da, 23,763.41 Da and 23,925.50 Da, Figure 5B). A molecular mass of 23,439.30 Da was set as the lowest, which was correlated to the theoretical mass of non-modified GST calculated from the sequence without any modifications (23,439.35 Da). The next mass close to the non-modified GST in the spectrum gave a characteristic shift of 163.05 Da which could suggest glycosylations (Figure 5B), however there was no noticeable glycosylation staining in the molecular mass range of GSTs in the *A. lumbricoides* extract (Figure 5C). Similar findings were obtained by enhanced chemiluminiscence assays confirming that the purified nGSTA was non-glycosylated (Figure 5D).

**Figure 6:**
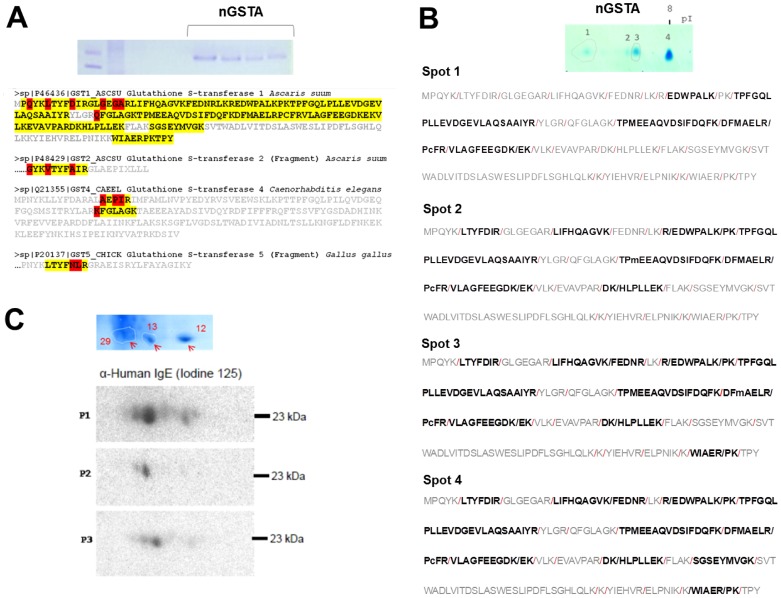
Polymorphic residues of the natural *A. lumbricoides* GST. (A) SDS-PAGE of the affinity purified nGSTA used for mass spectrometry and mapped peptides on the sequence of *A. suum* GST1 (UniProt P46436). Peptide matches are shown in yellow. Aminoacid positions with polymorphic residues are shown in their corresponding GST sequences (B) Two-dimensional electrophoresis of nGSTA and the mapped peptides from isoforms 1 to 4 on the sequence of *A. suum* GST1 (UniProt P46436). (C) Immunoblotting of the nGSTA with sera from Ascaris sensitized patients (n = 3) as primary antibodies. The relative positions of the GST isoforms after two-dimensional electrophoresis are presented in the upper panel.

To investigate the presence of potential amino acid substitutions, the nGSTA was separated by 12% SDS-PAGE and in-gel digested. The tryptic peptides were analyzed by Liquid Chromatography and Mass Spectrometry (LC-MS/MS). Twenty-eight peptides matched with the sequence of the GST1 of *A. suum* (UniProt P46436), resulting in 73.3% of sequence coverage (Figure 6A). In addition, peptides having amino acid substitutions were found to match GST sequences from other nematodes and vertebrates, being most of the polymorphic residues located at the N-terminal region. The nGSTA contained the peptide GYKVTYFAIR with three aminoacid substitutions at positions 3(Q→G), 6(L→V) and 10(D→A), which match to a small fragment of *A. suum* GST2 (Uniprot P48429). An alternative version of this peptide (LTYFNLR) was also detected with two amino acid substitutions at positions 10(D→N) and 11(I→L) that overlapped the sequence of *Gallus gallus* GST5 (Uniprot P20137). In addition, the peptide AEPIR with amino acid substitutions at positions 15(G→A), 17(G→P) and 18(A→I) and the peptide KFGLAGK with one amino-acid substitution at position 74(Q→K) were found to match the sequence of the nematode *Caenorhabditis elegans* GST4 (Figure 6A). Detailed information on the nGSTA peptides detected by LC-MS/MS is presented in the file S3. To further investigate the potential amino acid substitutions leading to different isoforms, the nGSTA was separated by isoelectric focusing using narrow-range strips (pI 4.0–8.0) followed by 12% SDS-PAGE. Four spots that were visible after Coomasie G250 staining were excised and in-gel digested. The tryptic peptides were separated by IP-RP-µHPLC and detected with a LTQ Orbitrap XL™ mass spectrometer. The coverage of the sequence of *A. suum* GST1 increased from one sample to another as the isoelectric point was closer to 8.0 as follows: 29.1% for spot 1; 41.7% for spot 2; 53.4% for spot 3 and 59.7% for spot 4 (Figure 6B). Interestingly, the spot 1 contained not only peptides with sequence homology to *A. suum* GST1 but also four peptides with sequence similarity to glutathione transferases of the π class in *Homo sapiens* and *Macaca mulatta* (file S4).

### IgE binding to nGSTA isoforms in the *Ascaris* extract

Two dimensional electrophoresis and LC-MS/MS analysis of the *A. lumbricoides* extract using strips with a broader range (pI 3–10) (Figure 6C), confirmed that coverage on the sequence of *A. suum* GST1 increased towards the most basic isoform (pI 8.0) and revealed the polymorphic peptide DILPVELAK between amino acid positions 120 to 128, which overlapped the sequence of *Wuchereria bancrofti* π class GST (Q86LL8) (file S5). Even though only 3 spots were visible and trypsin digested, the immunoblot using pooled sera from asthmatic patients showed that at least six GST isoforms bind IgE (Figure 6C). There were differences in the intensity of the IgE signals and the number of reactive spots according to the patient's sera; and one of the isoforms gave the most prominent IgE signal. It was not possible to define the trypsinized spots corresponding to each IgE binding isoform, but a superposition of the immunoblot signals with its gel replica suggests that spot 13 with a pI between 7.0 and 7.2, was the closest to the most intense IgE binding isoform. This spot harbored the polymorphic peptide GLAEPIR described in the GST of the canine heartworm *Dirofilaria immitis* (UniProt P46426) (file S5). Altogether these findings reveal that GSTA isoforms bind human IgE with different intensities.

## Discussion

The soil-transmitted nematode *A. lumbricoides* is well-known as a powerful inducer of Th2 immune responses and IgE synthesis in humans. There are several IgE binding components in the extract of Ascaris and their characterization is essential for better understanding the allergenicity of this nematode, as well as to define an array of cross-reactive and species-specific allergens for component resolved diagnosis of allergy in places where helminthiases are not yet controlled. In the whole extract of *A. suum*, we have previously observed an IgE-binding component of 23 kDa with sequence similarity to GST [6], but it was not defined if this molecule was an allergen. We here demonstrated that purified GSTs from *A. lumbricoides* have allergenic properties since they not only bind specific human IgE antibodies but also induce type-I hypersensitivity reactions in skin of sensitized subjects. Interestingly, the strength of the IgE reactivity to this molecule was greater than to mite and cockroach GSTs which have been previously recognized as allergens [10], [12]. In addition, we identified a group of asthmatic patients that were sensitized, not only to GSTA but also to mite GSTs (Der p 8 and Blo t 8), suggesting that this allergenic group might be of clinical relevance for some patients living in the tropics (Figure 2E & file S1). The International Union of Immunological Societies designated this allergen as Asc l 13.0101. Some studies have shown that helminth GSTs induce specific IgE response in humans [21], [23], [25] but the analysis on the impact of their allergenic properties is just at the beginning [25]. We have previously evaluated at the population level the allergenicity of Ascaris components such as tropomyosin (Asc l 3) [5]. The additional discovery of allergenic properties of GSTA may explain the strong IgE reactivity to mite extracts in populations living in the tropics and why the frequency of IgE sensitization to invertebrate GSTs is more frequent in the tropics than in populations living in temperate areas [17]. Furthermore, the characterization of GSTA as a new allergen of Ascaris will help in understanding the impact of helminth infection on the allergic sensitization, although further studies are needed to elucidate the predisposing factors for recognizing these GSTs and whether they contribute to the susceptibility to asthma and allergies in tropical environments.

We found a strong correlation between IgE levels to GSTA and other invertebrate GSTs (Figure 2C). Since the study population is co-exposed to all these allergenic sources, it is not feasible to know if these correlations with mite and cockroach GSTs have resulted from co-sensitization or cross-reactivity. However, the latter is likely to play a role because of the structural homology between Ascaris GSTs and other non-parasitic GSTs (Figure 3) that led to share potential epitopes (file S2). Indeed IgE antibody cross reactivity has been experimentally demonstrated between the GSTs from the nematode *W. bancrofti* and cockroach (Bla g 5) [25]. Also, immunization of rabbits with *A. lumbricoides* had shown to induce cross-reactive antibodies that recognize antigens in the extract of the mite *D. farinae* [27]. Older studies in our laboratory showed that 10% of non-immunotherapy-treated asthmatic patients had IgE response to *Schistosoma japonicum* GST [28], even though this helminth does not exist in our country. IgE cross-reactivity to GSTA or other invertebrate GST sources can explain these findings; however, considering that the strength of the IgE response is greater to GSTA than to mite and cockroach GSTs it is likely that *A. lumbricoides* is the primary sensitizer. Here, we were not able to detect cross-inhibition of IgE binding between Ascaris and mite GSTs, not even using depletion ELISAs, and a possible explanation may be the strong interference by high titers of competing IgG antibodies (data not shown).

The evaluation of a random sample set of asthmatics and controls revealed that at the population level the prevalence of positive IgE sensitization to GSTA was low. In addition, the strength of the IgE response to GSTs from Ascaris, mites and cockroach were consistently low. The same finding in the two sample datasets analyzed strongly support that this “*low reactivity*” is a hallmark of the IgE response to GSTA in adults. This was even more evident in asthmatic patients in which the strength of IgE levels to GSTA and cockroach GST (Bla g 5) where much lower compared to other non-GST allergens such as Der p 2 (Figure 4B). Since these patients were co-exposed to the allergenic sources (Ascaris, cockroach and *D. pteronyssinus*), we hypothesize that the low strength of IgE reactivity to GSTs is reflecting particular features of the humoral response to these molecules. Our findings are in agreement with previous studies reporting that IgE levels to allergenic GSTs are low [12] and raise many questions about the mechanisms underlying this weak IgE response to invertebrate GSTs in the context of exposure to a great variety of allergens. For instance, the IgE response to Bla g 5 has been described as the highest compared to any other cockroach allergen [10], [13], [29]. However, in this study the reactivity to Bla g 2 was higher than to Bla g 5 and in general the IgE response to cockroach is less intense than to mite allergens. Of relevance for clinical allergology, our results show that even though the frequency of IgE sensitization to GSTA is low, for some allergic patients living in the tropics, discrimination of the specific IgE response to GSTs might be necessary through component resolved diagnosis.

Since previous reports have described that human antibody responses to parasite GSTs may change with age [23], [30], we explore this relationship with the IgE response to GSTA. Interestingly, we found that the prevalence and the strength of specific IgE to GSTA decreased according to the age groups, children 7 to 13 years old having the highest rates of prevalence (35.2%) and the strongest IgE titers to GSTA. This could be explained by the fact that most Ascaris infections occur at early age. Nevertheless, it is also feasible that GSTA has intrinsic properties or different antigen abundance modulating this particular serological dynamic. The latter is suggested by IgE determinations on adult asthmatic patients from the same population in which the strength of specific IgE levels to Ascaris tropomyosin (Asc l 3) [5] were much higher than those observed in this study for GSTA.

We found that at least six GSTA isoforms bind IgE with different intensities (Figure 6C), in agreement with findings describing that helminth GSTs exist as isoforms and their amino acid substitutions and post-translational modifications such as glycosylations may influence their immunogenicity [20], [31]–[33]. Previous studies in *A. suum* have detected most of the GST expression in the brush border of the parasite's gut and weak to moderate expression in the cytoplasm of longitudinal muscle cells, oviducts and the lateral cords of the hypodermis [34]. To our knowledge, this study provides the first empirical evidence on GSTA isoforms at the protein level in the somatic extract of *A. lumbricoides*. Intact masses analysis detected four abundant isoforms and mass spectrometry analysis after trypsin digestion on the affinity purified nGSTA showed several amino acid substitutions, mainly located on the N-terminal end. The mass shift pattern of the isoforms compared to the monoisotopic mass of GSTA suggested glycosylations; however experiments to detect sugars found that GSTA is non glycosylated. Although we did not experimentally evaluate other potential post-translational modifications, the results support that GSTA isoforms are due to amino acid substitutions. In agreement with our observations, the draft genome of *A. suum* [35] contains at least 15 mapped sequences having similarity to GSTs on other nematodes but also in vertebrates (file S6). The presence of four tryptic peptides mapping to human π-class GSTs in the extract of *A. lumbricoides* was striking (file S4). However, genes with sequence homology to vertebrate GSTs have been also found in the genome of Ascaris (file S6). Due to the limited amounts of nGSTA in the somatic extract of *A. lumbricoides*, the peptide analyses were restricted to few spots that were visualized and excised from the gel. Nevertheless, immunoblotting analysis using sera from Ascaris sensitized patients confirmed that at least six isoforms that bind IgE do exists in this extract. In the field of helminth GST research the IgE binding properties are not routinely explored; for example eight GST isoforms were detected in *Necator americanus* using rabbit-immunized polyclonal antisera [33]. However, Mutapi *et al*., found two GST isoforms in *Schistosoma haematobium* that are specifically recognized by IgE from infected patients [23]. Further support of differential IgE binding at the isoform level have been obtained from studies in allergenic non-parasitic GSTs, for example, Jeong *et al*., found differences in the strength of IgE reactivity to two cockroach GST isoforms in allergic patients [19]. In addition, at least eight GST isoforms with differences in the intensity of antibody binding were found in the extract of *D. pteronyssinus* using anti-sera from mice immunized with rDer p 8 [12].

The results of this study, beyond its relevance for allergy diagnosis and the understanding of the allergenicity, provide important information to the field of helminth GST research. The finding that *A. lumbricoides* have GST isoforms with sequence similarity to those from vertebrates should be taken into account when designing tools aiming to target these molecules for anti-parasite therapy. On the other hand, since we observed that some individuals are able to produce high levels of specific IgE to GSTA (including children), the allergenic properties of GSTA should be considered when using helminth GSTs for vaccination; especially because *A. lumbricoides* is distributed worldwide, commonly found in poly-parasited subjects and different degrees of cross-reactivity between helminth GSTs have been described. However, the fact that natural GST isoforms have differential intensity in IgE binding could be considered for selecting natural or engineered non-allergenic vaccine candidates.

In summary, GSTA is a new allergen from *A. lumbricoides* of potential relevance for asthmatic patients predisposed to recognize invertebrate pan-allergens and co-sensitized to mite and cockroach GSTs. As has been described for other allergenic GSTs, the strength of IgE reactivity to GSTA is low and the prevalence and IgE levels to GSTA are highest during childhood. GSTA has several isoforms with differential IgE recognition.

## Materials and Methods

### Ethical statement

This study was conducted following the ethical principles for medical research stated in the Declaration of Helsinki. The Bioethics Committee of the University of Cartagena approved the study. A full verbal explanation of the investigation was given to each participant and written informed consent was obtained from all subjects or their parents or legal guardians.

### Sera

Sera were obtained from the repository of the Institute for Immunological Research (University of Cartagena, Colombia). To evaluate the IgE binding capacity of purified GSTs from Ascaris and the house dust mites *B. tropicalis* and *D. pteronyssinus*, we tested sera from 26 asthmatic patients and 3 non-asthmatic controls with positive IgE sensitization to Ascaris (Mean age 27±13.3 years). This set of sera plus 7 Ascaris negative sera were used to study the correlation between the IgE levels to purified GSTs from Ascaris and mites (n = 36, file S1). The frequency of IgE reactivity to the recombinant Ascaris GST (rGSTA) was estimated in an independent cohort of asthmatic patients (n = 215) and controls (n = 91) living in Cartagena (Colombia). These sera were randomly selected regardless of Ascaris sensitization and include individuals from 7 to 70 years of age (Mean age 31.8±16.1 years). Asthmatic patients were recruited in health care centers and were representative of the lowest strata in the population (1 to 3) as described previously [36]. Non-asthmatic controls share the same sociodemographic characteristics of the patients. Ascaris infection has been previously documented in this environment and eggs have been detected in stool samples early in life [37].

### Purification of natural *A. lumbricoides* GST

*A. lumbricoides* extract was obtained as described previously [5]. To purify the natural Ascaris GST (nGSTA), the extract was reconstituted in 1x PBS pH 7.3 and incubated during 1 hour with Glutathione-Sepharose 4B (GE Healthcare). After 3 washes with PBS, the affinity bound GSTs were eluted with 10 mM of L- glutathione reduced (Cat G4251, Sigma, St. Louis), diluted in 50 mM Tris-HCl pH 8.0. Proteins were dialyzed during 16 hours (6000 Da cut-off membrane, Spectrum Laboratories). The same method was used to purify the natural GSTs of *B. tropicalis* (nBlo t 8). The eluates containing the purified GSTs were visualized in denaturing 12% SDS-PAGE gels after Coomassie staining.

### Cloning and purification of recombinant *Ascaris* GST, mite and cockroach GSTs

Recombinant GST1 from *A. suum* (rGSTA) was cloned into the pQE30 vector based on the sequence deposited at GenBank under accession number (X75502.1). The protein was expressed in *E. coli* M15 and purified as a 6xHis-tagged protein. The recombinant GST of *B. tropicalis* (rBlo t 8), was amplified from a cDNA library of *B. tropicalis* [38], and the fragment was cloned into a pET100 vector and expressed in BL21 (DE3) cells. Recombinant *D. pteronyssinus* GSTmu (rDer p 8) (AY825938) and Der p 2 (ABG76196) were subcloned into the pET45b+ vector and expressed in the Origami BL21 (DE3) bacterial system. Induced cultures were re-suspended in native buffer (50 mM NaH_2_PO_4_, 300 mM NaCl), incubated with lysozyme (1 mg/ml, 30 minutes on ice) and sonicated. Lysates were incubated with Ni-NTA resin (Invitrogen) for one hour, washed with native buffer plus 20 mM imidazole, and eluted with native buffer plus 250 mM imidazole as a 6xHis-tagged protein. The Ascaris allergens rABA-1 and rAsc l 3 were produced as previously described [5], [6]. Recombinant cockroach allergens were obtained from Indoor Biotechnologies, Inc. (Charlottesville, VA) and expressed in *E coli* (Bla g 5) or *Pichia pastoris* (Bla g 2) as previously described [10], [39].

### ELISA

The prevalence of specific IgE antibodies against nGSTA and rGSTA were determined by indirect ELISA. Appropriate antigen concentrations were obtained by titration. Briefly, each well of a 96-well plate was coated with 0.5 µg of nGSTA and 0.75 µg of rGSTA diluted in PBS (pH 7.4), incubated overnight at 4°C, washed with 0.1%-Tween-20/PBS (TPBS) and blocked during 3 hours with 3% bovine serum albumin. Sera samples diluted 1∶5 in blocking buffer were added and incubated overnight, washed and incubated with 100 µl of anti-human IgE Alkaline-Phosphatase Conjugate (Sigma). The assay was developed with *ρ*-nitrophenylphosphate substrate (1 mg/mL, Sigma) and the enzymatic reaction stopped after 30 minutes of substrate incubation by adding 3 N NaOH. Absorbance was measured at 405 nm using a Spectrophotometer (Spectra MAX 250 Molecular Device Sunnyvale, California 94089). All the determinations were done by duplicate and IgE levels above 0.130 OD_405 nm_ were considered positive (mean OD of 7 non-Ascaris sensitized subjects +3 SD). Specific IgE levels to nBlo t 8 (0.5 µg), rBlot 8 (0.75 µg), rDer p8 (0.75 µg), rBla g 5 (0.75 µg), rBla g 2 (0.75 µg) and rDer p 2 (1 µg) were measured by ELISA as aforementioned. Positive and negative control sera were included in each plate.

### Skin tests

The ability of nGSTA and nBlo t 8 to induce mast cell degranulation was evaluated by skin prick tests in eight asthmatic patients with a positive IgE result to the rGSTA [OD >0.130], two parasited children, and two asthmatic patients with a negative IgE result to the rGSTA, who provided consent to perform the procedure (Table 1). The purified allergens were prepared in glycerol at 0.025 µg/µl for nGSTA and nBlo t 8. The Ascaris extract was prepared at 0.15 µg/µl. Mite extracts were kindly donated by Leti Laboratories (Madrid, Spain). Histamine phosphate (10 mg/mL) was used as positive control and glycerol as negative control. The test was positive if the mean diameter of the wheal was >3 mm at 15 minutes.

### Structural homology

Homology-based molecular modeling of *A. suum* GST, Bla g 5 (O18598), Blo t 8 (ACV04860) and Der p 8 (P46419) structures were done in the SWISS-MODEL server using as templates the experimentally defined structures 2on5H (*O. volvulus*), 1m0uA (*D. melanogaster*), 1gsuB (*G. gallus*) and 1c72d (*G. gallus*), respectively. Models were refined in Deep-View (energy minimization and rotamer replacements). Their quality was evaluated by several tools, including Ramachandran plots, WHATIF, QMEAN4 index and energy values (GROMOS96 force field). Relative values of accessible solvent area (r-ASA) were determined by ASA-view [40]. The rGSTA sequence was then aligned to each allergenic sequence to identify conserved residues. Those conserved and solvent accessible residues (rASA >0.25) were then located in the 3D-model to identified clustered areas (>4 residues) as possible cross-reactive antigen binding areas.

### HPLC separation of *A. lumbricoides* extract

The separation of the *A. lumbricoides* extract was done by high-performance liquid chromatography (UltiMate 3000 nano-HPLC system, Dionex Part of Thermo Fisher Scientific, Amsterdam, The Netherlands), using the following conditions: columns: 200×0.1 mm I.D. PS-DVB monolith column; 10×0.2 mm I.D. monolithic PS-DVB trap column; eluent A: water +0.05% trifluoroacetic acid; eluent B: acetonitrile +0.05% trifluoroacetic acid; 3 min trapping with 0.10% aqueous heptafluorobutyric acid (10 µl/min); temperature: 55°C; flow rate: 1 µl/min; gradient: 5–60% B in 60 min; injection of 1.0 µl of 60.5 µg/ml protein extract. The PS-DVB monolith and monolithic PS-DVB trap column were prepared at Department of Molecular Biology, Division of Chemistry and Bioanalytics, University of Salzburg, Salzburg, Austria and are commercially available as ProSwift® reversed-phase monolith columns from Thermo Fisher Scientific, Vienna, Austria. Mass spectrometry analyses were performed using an electrospray ionization – time-of-flight mass spectrometer (MicrOTOF, Bruker, Bremen, Germany).

### HPLC-FT-MS analysis for intact masses

The separation of nGSTA was carried out using ProSwift® PS-DVB monoliths (Dionex Part of Thermo Fisher Scientific, Amsterdam, The Netherlands) with a length of 50 mm and an inner diameter of 1 mm in an HPLC system with low pressure gradient formation (Accela™, ThermoFisher Scientific, Bremen, Germany) using the following set-up: eluent A: 0.05% aqueous trifluoroacetic acid; eluent B: acetonitrile +0.05% tri-fluoroacetic acid; gradient: 20–50% B in 45 min; flow rate: 60 µl/min; temperature: 60°C; injection volume: 15 µl. The HPLC system was hyphenated to a linear ion trap - Orbitrap mass spectrometer (LTQ-Orbitrap-XL, Thermo Fisher Scientific, Bremen, Germany) equipped with an Ion Max electrospray ionization source with a 34-gauge metal needle (both Thermo Fisher Scientific). Electrospray ionization was conducted in positive mode. Data were acquired with a resolution of 100,000 in a mass range of 700–3,500 m/z. For data acquisition and evaluation, the Xcalibur 2.0.7 software (Thermo Fisher Scientific) was used. Mass spectra were deconvoluted with the Xtract software (Thermo Fisher Scientific).

### Two-dimensional electrophoresis

The Ascaris extract (200 µg) and the purified nGSTA (25–50 µg) were separated by 2D-SDS-PAGE as previously described [5]. Proteins were precipited with the ProteoExtract® Protein Precipitation Kit (Calbiochem, San Diego, USA). Pellets were reconstituted in isoelectric focusing (IEF) buffer (8 M urea, 4% chaps, 0.0002% Bromophenol Blue, 50 mM DTT, 0.2% BioLyte Carrier Ampholytes pH 3–10) and the IEF performed in immobilized gradients of pI 3–10 (for the Ascaris extract) and pI 4.0–8.0 (for the purified nGSTA) (ReadyStrip IPG Strips, Protean IEF system; Bio-Rad, Hercules, CA, USA).

### In-gel digestion

The three visible spots resulting from the two-dimensional separation of the *A. lumbricoides* extract and Coomassie G250-staining were excised and in-gel digested using the ProteoExtract® All-In-One Trypsin Digestion Kit (Calbiochem, San Diego, USA). Besides, four spots resulting of the two-dimensional separation of the nGSTA were excised from the gel and digested as follows: pieces containing the spots of the nGSTA were washed with MilliQ water and 100 mM ammonium bicarbonate buffer (ABC-buffer), then unstained with acetonitrile (ACN) and dehydrated at 30°C with a concentrator (Eppendorf, Hamburg, Germany). The reduction was performed at 56°C for 30 min with 10 mM dithiothreitol in 100 mM ABC-buffer and the alkylation was done at room temperature for 20 min with 55 mM iodacetamide in 100 mM ABC-buffer. After washing with ABC-buffer and acetonitrile the slices were dried again at 30°C in the concentrator. The digestion was accomplished as prescribed with sequencing grade modified Trypsin (#V5111; Promega, Madison, USA). The peptides were extracted from the gel slices with buffer (90% acetonitrile, 0.35 mM ABC, 0.5% formic acid) and concentrated to a final volume of 100 µl.

### HPLC and MS analysis of in-gel digested samples

Tryptic peptides were sequenced after separation by ion-pair reversed-phase capillary high-performance liquid chromatography (HPLC) directly coupled to an electrospray ionization-quadrupole-time-of-flight mass spectrometer (Q-Tof Ultima Global; Waters) as previously described [5]. Because of its high mass accuracy, a high-resolution Orbitrap mass spectrometer was used to sequence the tryptic peptides of the spots excised from the purified nGSTA [26]. Briefly, peptides were separated by capillary-HPLC (Model UltiMate3000, Dionex Part of Thermo Fisher Scientific, Amsterdam, The Netherlands) equipped with a 3 nL z-shaped capillary detection cell. Separations were generally accomplished in monolithic 150×0.20 mm PS-DVB capillary columns (prepared at the Department of Molecular Biology, Division of Chemistry and Bioanalytics, University of Salzburg, Salzburg, Austria) and at 55°C with gradients of acetonitrile in 0.05% aqueous trifluoroacetic acid (TFA) at flow rates of 1 µl/min. MS analysis was performed with a linear ion trap-Orbitrap mass spectrometer (Model LTQ-Orbitrap-XL, Thermo Fisher Scientific). A nano electrospray source was utilized with a 20 µm id fused silica capillary and a tip drawn to 10 µm id (New Objective, Woburn, USA). Mass calibration was done with the commercially available positive calibration solution for LTQ XL and LTQ Hybrids (Sigma Aldrich, St. Louis, MO, USA). The instrument was operated in positive electrospray ionization mode with a spray voltage of 1.8 kV, a capillary voltage of 36.0 V and a capillary temperature of 200°C. The MS parameters were optimized in the range of m/z 400–2000 by infusing a solution of glu-1-fibrinopeptide B (Sigma Aldrich, St. Louis, MO, USA) with a concentration of 106 fmol/µl at a resolution of 60 000 at m/z 400. A data dependent MS/MS measurement was performed. After the full scan in the Orbitrap mass analyzer, the three most intensive ions were isolated and fragmented in the ion trap with collision induced fragmentation (CID) with 35% normalized collision energy and an isolation window of ±1.0 Da. Dynamic exclusion for 60 seconds was used to prevent multiple fragmentation of the same precursor ion. For each sample, three replicates were measured and analyzed with the Mascot search engine 2.2 (Matrix Science, London, UK) and the software Proteome Discoverer 1.1 (Thermo Fisher Scientific). The following parameters were used for peptide identification: taxonomy, all entries; variable modification, methionine oxidation; fixed modification, carbamidomethyl (C); enzyme, trypsin; peptide tolerance, ±10 ppm; MS/MS tolerance, ±0.3 Da; maximum missed cleavages, 1. Samples were first searched against a database containing all proteins of the order “Ascaridida” (around 12 000 proteins). Once confirmed that no other molecules overlap with the 2D separation parameters of GSTA, we search against species-specific UniProt/SwissProt GST databases containing a broader variety of GSTs (293 proteins) homologs in vertebrate and invertebrates.

### Two dimensional immunoblotting

For immunoblotting, proteins were electro-transferred onto nitrocellulose membranes, blocked and washed. After overnight incubation with sera from Ascaris sensitized patients, membranes were washed and bound IgE was detected using [^125^I] - labeled α-human IgE (MedPro, Vienna, Austria). Radiographic signals were visualized by overnight exposure to a phosphorimager screen and subsequent scanning using a Fujifilm BAS-1800 II reader. Specific IgE to the Ascaris extract and HPLC fractions was detected using patient's sera as primary antibody and alkaline phosphatase-labeled anti IgE (αIgE-AP) as secondary antibody (Clone GE-1, Sigma, Cat A3076).

### Glycosylation analysis

Glycodetection of the Ascaris extracts was performed using the PAS staining, which reveals the aldehyde groups that are exposed when the sugar rings are open. Briefly proteins were electrotransferred to PVDF membranes at 15V during 50 minutes, washed with distilled water, incubated in a solution of 1% periodic acid/3% acetic acid during 15 min; then, the membranes were washed 3 times with distilled water (5 minutes), incubated 15 minutes with the Schiff's reagent (Fuchsin sulfite) in a dark chamber without agitation and washed 3 times with distilled water. Glycosilation signals were visualized on the membranes. For nGSTA and nBlo t 8, the metaperiodate method was used. Briefly, PVDF-electrotransferred proteins were incubated in the dark for 20 minutes in a solution of 10 mM sodium metaperiodate dissolved in 100 mM acetate buffer (pH 5.5). After washed, the membrane was incubated for 60 minutes at RT with 0.125 mM biotin hydrazide (diluted 1∶5000 in 100 mM acetate buffer). Streptavidine-horseradish peroxidase (1∶100.000 diluted in PBS) was added and incubated during 30 minutes. Membranes were exposed to chemiluminiscence reagents (Thermo Scientific, Rockford, IL, USA) and images were captured with a CCD camera (GBOX; Syngene, Cambridge, UK).

### Statistics

IgE levels to purified allergens were expressed as the mean OD (optical density) of duplicate wells. Non-parametric paired Wilcoxon signed-rank test was used to compare the strength of IgE levels to GSTs. The relationship between IgE levels to GSTs were calculated by Spearman rank correlation. The comparison between the frequency of IgE sensitization between cases and controls was done by χ^2^ analysis and adjusted by covariates using logistic regression. The comparison of IgE levels to rGSTA between asthmatic patients and controls was estimated using the non-parametric Mann Whitney U test. To evaluate the effect of age on the IgE levels to rGSTA, the study participants were stratified in five groups (7–13 years, n = 62), (14–27 years, n = 66), (28–37 years, n = 61), (38–48 years, n = 60), (49–70 years, n = 57) as defined by the 20^th^, 40th, 60^th^ and 80^th^ percentile of the age. Linear regressions were used to analyze the effect of age, gender and disease status on the IgE levels to rGSTA. All statistical analyses were two-tailed and the significance was set at p<0.05. Analyses were performed using IBM Statistics SPSS v20 (IBM Corp) and GraphPhad Prism.

## Supporting Information

File S1 **Descriptive of specific IgE levels to purified Ascaris and mite GSTs (n = 36).**
(PDF)

File S2 **Multiple sequence analysis between parasitic and non-parasitic GST plus the amino acid composition of the patches with surface-exposed residues between Ascaris GST1 and non-parasitic GSTs from mites (Der p 8, Blo t 8) and cockroach (Bla g 5).**
(PDF)

File S3 **Tryptic peptides coinciding with known GST sequences after LC-MS/MS of purified nGSTA.**
(DOCX)

File S4 **Peptides identified in four spots containing nGSTA after 2D electrophoresis and LTQ Orbitrap XL mass spectrometry analysis.**
(XLS)

File S5 **Peptides identified in three spots containing nGSTA after 2D electrophoresis and LC-MS/MS analysis.**
(PDF)

File S6 **Sequences with homology to GSTs in the draft genome of** ***A. suum*** **.**
(XLSX)

## References

[1] Haahtela T, Holgate S, Pawankar R, Akdis C, Benjaponpitak S, et al (2013) The biodiversity hypothesis and allergic disease: World Allergy Organization position statement. World Allergy Organization Journal 6: 3. 23663440 10.1186/1939-4551-6-3 PMC3646540

[2] Acevedo N, Caraballo L (2011) IgE cross-reactivity between Ascaris lumbricoides and mite allergens: possible influences on allergic sensitization and asthma. Parasite Immunol 33: 309–321. 21388422 10.1111/j.1365-3024.2011.01288.x

[3] Cooper PJ (2009) Interactions between helminth parasites and allergy. Curr Opin Allergy Clin Immunol 9: 29–37. 19106698 10.1097/ACI.0b013e32831f44a6 PMC2680069

[4] Caraballo L, Acevedo N (2011) New Allergens of Relevance in Tropical Regions: The Impact of Ascaris lumbricoides Infections. World Allergy Organ J 4: 77–84. 23282442 10.1097/WOX.0b013e3182167e04 PMC3651106

[5] Acevedo N, Erler A, Briza P, Puccio F, Ferreira F, et al (2011) Allergenicity of Ascaris lumbricoides tropomyosin and IgE sensitization among asthmatic patients in a tropical environment. Int Arch Allergy Immunol 154: 195–206. 20861641 10.1159/000321106

[6] Acevedo N, Sanchez J, Erler A, Mercado D, Briza P, et al (2009) IgE cross-reactivity between Ascaris and domestic mite allergens: the role of tropomyosin and the nematode polyprotein ABA-1. Allergy 64: 1635–1643. 19624559 10.1111/j.1398-9995.2009.02084.x

[7] Nakazawa T, Khan AF, Yasueda H, Saito A, Fukutomi Y, et al (2013) Immunization of rabbits with nematode Ascaris lumbricoides antigens induces antibodies cross-reactive to house dust mite Dermatophagoides farinae antigens. Biosci Biotechnol Biochem 77: 145–150. 23291773 10.1271/bbb.120626

[8] Santos AB, Rocha GM, Oliver C, Ferriani VP, Lima RC, et al. (2008) Cross-reactive IgE antibody responses to tropomyosins from Ascaris lumbricoides and cockroach. J Allergy Clin Immunol 121: : 1040–1046 e1041. 10.1016/j.jaci.2007.12.1147 18275995

[9] Sheehan D, Meade G, Foley VM, Dowd CA (2001) Structure, function and evolution of glutathione transferases: implications for classification of non-mammalian members of an ancient enzyme superfamily. Biochem J 360: 1–16. 11695986 10.1042/0264-6021:3600001 PMC1222196

[10] Arruda LK, Vailes LD, Platts-Mills TA, Hayden ML, Chapman MD (1997) Induction of IgE antibody responses by glutathione S-transferase from the German cockroach (Blattella germanica). J Biol Chem 272: 20907–20912. 9252418 10.1074/jbc.272.33.20907

[11] Shankar J, Singh BP, Gaur SN, Arora N (2006) Recombinant glutathione-S-transferase a major allergen from Alternaria alternata for clinical use in allergy patients. Mol Immunol 43: 1927–1932. 16430961 10.1016/j.molimm.2005.12.006

[12] Huang CH, Liew LM, Mah KW, Kuo IC, Lee BW, et al (2006) Characterization of glutathione S-transferase from dust mite, Der p 8 and its immunoglobulin E cross-reactivity with cockroach glutathione S-transferase. Clin Exp Allergy 36: 369–376. 16499649 10.1111/j.1365-2222.2006.02447.x

[13] Satinover SM, Reefer AJ, Pomes A, Chapman MD, Platts-Mills TA, et al (2005) Specific IgE and IgG antibody-binding patterns to recombinant cockroach allergens. J Allergy Clin Immunol 115: 803–809. 15806002 10.1016/j.jaci.2005.01.018

[14] O'Neill GM, Donovan GR, Baldo BA (1995) Glutathione S-transferase a major allergen of the house dust mite, Dermatophagoides pteronyssinus. Immunol Lett 48: 103–107. 8719107 10.1016/0165-2478(95)02452-2

[15] Hales BJ, Martin AC, Pearce LJ, Laing IA, Hayden CM, et al (2006) IgE and IgG anti-house dust mite specificities in allergic disease. J Allergy Clin Immunol 118: 361–367. 16890759 10.1016/j.jaci.2006.04.001

[16] Patelis A, Gunnbjornsdottir M, Malinovschi A, Matsson P, Onell A, et al. (2012) Population-based study of multiplexed IgE sensitization in relation to asthma, exhaled nitric oxide, and bronchial responsiveness. J Allergy Clin Immunol 130: : 397–402 e392. 10.1016/j.jaci.2012.03.046 22633327

[17] Weghofer M, Thomas WR, Kronqvist M, Mari A, Purohit A, et al (2008) Variability of IgE reactivity profiles among European mite allergic patients. Eur J Clin Invest 38: 959–965. 19021722 10.1111/j.1365-2362.2008.02048.x

[18] Ebo DG, Bridts CH, Verweij MM, De Knop KJ, Hagendorens MM, et al (2010) Sensitization profiles in birch pollen-allergic patients with and without oral allergy syndrome to apple: lessons from multiplexed component-resolved allergy diagnosis. Clin Exp Allergy 40: 339–347. 19709127 10.1111/j.1365-2222.2009.03345.x

[19] Jeong KY, Jeong KJ, Yi MH, Lee H, Hong CS, et al (2009) Allergenicity of sigma and delta class glutathione S-transferases from the German cockroach. Int Arch Allergy Immunol 148: 59–64. 18716404 10.1159/000151506

[20] Sommer A, Nimtz M, Conradt HS, Brattig N, Boettcher K, et al (2001) Structural analysis and antibody response to the extracellular glutathione S-transferases from Onchocerca volvulus. Infect Immun 69: 7718–7728. 11705953 10.1128/IAI.69.12.7718-7728.2001 PMC98867

[21] Hong SJ, Yun Kim T, Gan XX, Shen LY, Sukontason K, et al (2002) Clonorchis sinensis: glutathione S-transferase as a serodiagnostic antigen for detecting IgG and IgE antibodies. Exp Parasitol 101: 231–233. 12594964 10.1016/s0014-4894(02)00112-1

[22] Auriault C, Gras-Masse H, Pierce RJ, Butterworth AE, Wolowczuk I, et al (1990) Antibody response of Schistosoma mansoni-infected human subjects to the recombinant P28 glutathione-S-transferase and to synthetic peptides. J Clin Microbiol 28: 1918–1924. 2121788 10.1128/jcm.28.9.1918-1924.1990 PMC268078

[23] Mutapi F, Bourke C, Harcus Y, Midzi N, Mduluza T, et al (2011) Differential recognition patterns of Schistosoma haematobium adult worm antigens by the human antibodies IgA, IgE, IgG1 and IgG4. Parasite Immunol 33: 181–192. 21204849 10.1111/j.1365-3024.2010.01270.x PMC3084999

[24] Al-Sherbiny M, Osman A, Barakat R, El Morshedy H, Bergquist R, et al (2003) In vitro cellular and humoral responses to Schistosoma mansoni vaccine candidate antigens. Acta Trop 88: 117–130. 14516923 10.1016/s0001-706x(03)00195-5

[25] Santiago HC, LeeVan E, Bennuru S, Ribeiro-Gomes F, Mueller E, et al. (2012) Molecular mimicry between cockroach and helminth glutathione S-transferases promotes cross-reactivity and cross-sensitization. J Allergy Clin Immunol 130: : 248–256 e249. 10.1016/j.jaci.2012.02.045 PMC3387355 22541242

[26] Mohr J, Swart R, Samonig M, Bohm G, Huber CG (2010) High-efficiency nano- and micro-HPLC—high-resolution Orbitrap-MS platform for top-down proteomics. Proteomics 10: 3598–3609. 20859959 10.1002/pmic.201000341

[27] Nakazawa T, Khan AF, Yasueda H, Saito A, Fukutomi Y, et al (2013) Immunization of rabbits with nematode Ascaris lumbricoides antigens induces antibodies cross-reactive to house dust mite Dermatophagoides farinae antigens. Biosci Biotechnol Biochem 77: 145–150. 23291773 10.1271/bbb.120626

[28] Jimenez SP, Mendoza L, Caraballo D, Chua L (2007) IgE antibody responses to recombinant allergens of Blomia tropicalis and Dermatophagoides pteronyssinus in a tropical environment. Allergy and Clinical Immunology International 19: 233–238.

[29] Arruda LK, Vailes LD, Mann BJ, Shannon J, Fox JW, et al (1995) Molecular cloning of a major cockroach (Blattella germanica) allergen, Bla g 2. Sequence homology to the aspartic proteases. J Biol Chem 270: 19563–19568. 7642642 10.1074/jbc.270.33.19563

[30] Salinas G, Sinha K, Cooper JP, Whitworth JA, Taylor DW (1996) Human isotype antibody responses to an Onchocerca volvulus glutathione S-transferase. Parasite Immunol 18: 377–386. 9229391 10.1046/j.1365-3024.1996.d01-124.x

[31] Liebau E, Hoppner J, Muhlmeister M, Burmeister C, Luersen K, et al (2008) The secretory omega-class glutathione transferase OvGST3 from the human pathogenic parasite Onchocerca volvulus. FEBS J 275: 3438–3453. 18537826 10.1111/j.1742-4658.2008.06494.x

[32] Perbandt M, Hoppner J, Betzel C, Walter RD, Liebau E (2005) Structure of the major cytosolic glutathione S-transferase from the parasitic nematode Onchocerca volvulus. J Biol Chem 280: 12630–12636. 15640152 10.1074/jbc.M413551200

[33] Zhan B, Perally S, Brophy PM, Xue J, Goud G, et al (2010) Molecular cloning, biochemical characterization, and partial protective immunity of the heme-binding glutathione S-transferases from the human hookworm Necator americanus. Infect Immun 78: 1552–1563. 20145100 10.1128/IAI.00848-09 PMC2849424

[34] Liebau E, Eckelt VH, Wildenburg G, Teesdale-Spittle P, Brophy PM, et al (1997) Structural and functional analysis of a glutathione S-transferase from Ascaris suum. Biochem J 324 (Pt 2): 659–666. 10.1042/bj3240659 PMC1218479 9182731

[35] Jex AR, Liu S, Li B, Young ND, Hall RS, et al (2011) Ascaris suum draft genome. Nature 479: 529–533. 22031327 10.1038/nature10553

[36] Acevedo N, Mercado D, Vergara C, Sanchez J, Kennedy MW, et al (2009) Association between total immunoglobulin E and antibody responses to naturally acquired Ascaris lumbricoides infection and polymorphisms of immune system-related LIG4, TNFSF13B and IRS2 genes. Clin Exp Immunol 157: 282–290. 19604268 10.1111/j.1365-2249.2009.03948.x PMC2730854

[37] Acevedo N, Sanchez J, Zakzuk J, Bornacelly A, Quiroz C, et al (2012) Particular characteristics of allergic symptoms in tropical environments: follow up to 24 months in the FRAAT birth cohort study. BMC Pulm Med 12: 13. 22439773 10.1186/1471-2466-12-13 PMC3331807

[38] Caraballo L, Avjioglu A, Marrugo J, Puerta L, Marsh D (1996) Cloning and expression of complementary DNA coding for an allergen with common antibody-binding specificities with three allergens of the house dust mite Blomia tropicalis. J Allergy Clin Immunol 98: 573–579. 8828535 10.1016/s0091-6749(96)70091-x

[39] Li M, Gustchina A, Alexandratos J, Wlodawer A, Wunschmann S, et al (2008) Crystal structure of a dimerized cockroach allergen Bla g 2 complexed with a monoclonal antibody. J Biol Chem 283: 22806–22814. 18519566 10.1074/jbc.M800937200 PMC2504871

[40] Ahmad S, Gromiha M, Fawareh H, Sarai A (2004) ASAView: database and tool for solvent accessibility representation in proteins. BMC Bioinformatics 5: 51. 15119964 10.1186/1471-2105-5-51 PMC420234

