# Supplementary material for: Proteomic and Immunochemical Characterization of Glutathione Transferase as a New Allergen of the Nematode Ascaris lumbricoides
Source: PLoS One. 2013 Nov 4;8(11):e78353. doi: 10.1371/journal.pone.0078353 (PMC3817249; doi:10.1371/journal.pone.0078353)
Supplement: File S1 — Descriptive of specific IgE levels to purified Ascaris and mite GSTs (n = 36). (PDF) [file pone.0078353.s001.pdf]

Supplementary file S1. Descriptive of specific IgE levels to purified Ascaris and mite GSTs (n = 36)

						Specific IgE levels (OD units)				
**#**	**Sample ID.**	**Gender**	**Age**	**IgE+ (As)**	**Group**	**rGSTA**	**nGSTA**	**rBlo t 8**	**nBlo t 8**	**rDer p 8**
1	A1073	F	23	Yes	Asthma	0.242	0.301	0.172	0.271	0.259
2	A299	F	41	Yes	Asthma	0.118	0.146	0.116	0.132	0.152
3	A323	M	48	Yes	Asthma	0.201	0.312	0.168	0.207	0.241
4	A352	F	9	Yes	Asthma	0.192	0.332	0.15	0.34	0.197
5	A368	M	44	Yes	Asthma	0.252	0.38	0.179	0.307	0.274
6	A386	F	37	Yes	Asthma	0.122	0.201	0.098	0.147	0.115
7	A392	M	10	Yes	Asthma	0.185	0.38	0.134	0.213	0.188
8	A393	F	16	Yes	Asthma	0.201	0.312	0.168	0.207	0.241
9	A448	F	30	Yes	Asthma	0.485	1.571	0.24	0.946	0.634
10	A485	F	26	Yes	Asthma	0.162	0.35	0.12	0.199	0.264
11	A489	F	10	Yes	Asthma	0.148	0.179	0.111	0.168	0.142
12	A492	F	30	Yes	Asthma	0.142	0.182	0.115	0.139	0.181
13	A519	M	8	Yes	Asthma	0.127	0.2	0.142	0.166	0.127
14	A671	M	47	Yes	Asthma	0.101	0.213	0.087	0.108	0.147
15	Asc067	F	29	Yes	Asthma	0.117	0.147	0.105	0.127	0.159
16	Asc105	F	23	Yes	Asthma	0.112	0.135	0.107	0.103	0.081
17	Asc112	F	28	Yes	Asthma	0.153	0.104	0.092	0.091	0.125
18	Asc114	M	9	Yes	Asthma	0.191	0.136	0.083	0.089	0.089
19	Asc125	F	42	Yes	Asthma	0.113	0.136	0.108	0.113	0.099
20	Asc146	M	7	Yes	Asthma	0.105	0.174	0.109	0.164	0.114
21	Asc151	F	21	Yes	Asthma	0.262	0.415	0.111	0.137	0.107
22	Asc159	F	30	Yes	Asthma	0.142	0.113	0.089	0.104	0.116
23	Asc227	F	35	Yes	Asthma	0.137	0.116	0.109	0.104	0.114
24	Asc236	F	21	Yes	Asthma	0.13	0.144	0.103	0.149	0.115
25	Asc285	F	47	Yes	Asthma	1.122	0.306	0.261	0.284	0.345
26	Asc290	F	38	Yes	Asthma	0.52	0.164	0.156	0.129	0.218
27	CAR271	n.a	n.a	Yes	Control	0.205	0.303	0.14	0.2	0.231
28	CAR799	F	35	Yes	Control	0.235	0.269	0.176	0.25	0.239
29	CAR950	F	40	Yes	Control	0.129	0.181	0.103	0.147	0.138
30	A415	F	27	No	Asthma	0.089	0.103	0.085	0.087	0.095
31	A480	F	48	No	Asthma	0.119	0.102	0.087	0.095	0.104
32	A498	F	62	No	Asthma	0.092	0.12	0.087	0.121	0.102
33	Asc043	F	8	No	Asthma	0.077	0.095	0.076	0.081	0.117
34	Asc071	M	11	No	Asthma	0.086	0.079	0.077	0.076	0.071
35	Asc164	F	26	No	Asthma	0.106	0.1	0.085	0.099	0.094
36	CAR272	n.a	n.a	No	Control	0.102	0.092	0.084	0.084	0.086
