# Supplementary material for: Proteomic and Immunochemical Characterization of Glutathione Transferase as a New Allergen of the Nematode Ascaris lumbricoides
Source: PLoS One. 2013 Nov 4;8(11):e78353. doi: 10.1371/journal.pone.0078353 (PMC3817249; doi:10.1371/journal.pone.0078353)
Supplement: File S2 — Multiple sequence analysis between parasitic and non-parasitic GST plus the amino acid composition of the patches with surface-exposed residues between Ascaris GST1 and non-parasitic GSTs from mites (Der p 8, Blo t 8) and cockroach (Bla g 5). (PDF) [file pone.0078353.s002.pdf]

Supplementary file S2. Multiple sequence analysis between parasitic and non-parasitic GST plus the amino acid composition of the patches with surface-exposed residues between Ascaris GST1 and non-parasitic GSTs from mites (Der p 8, Blo t 8) and cockroach (Bla g 5).

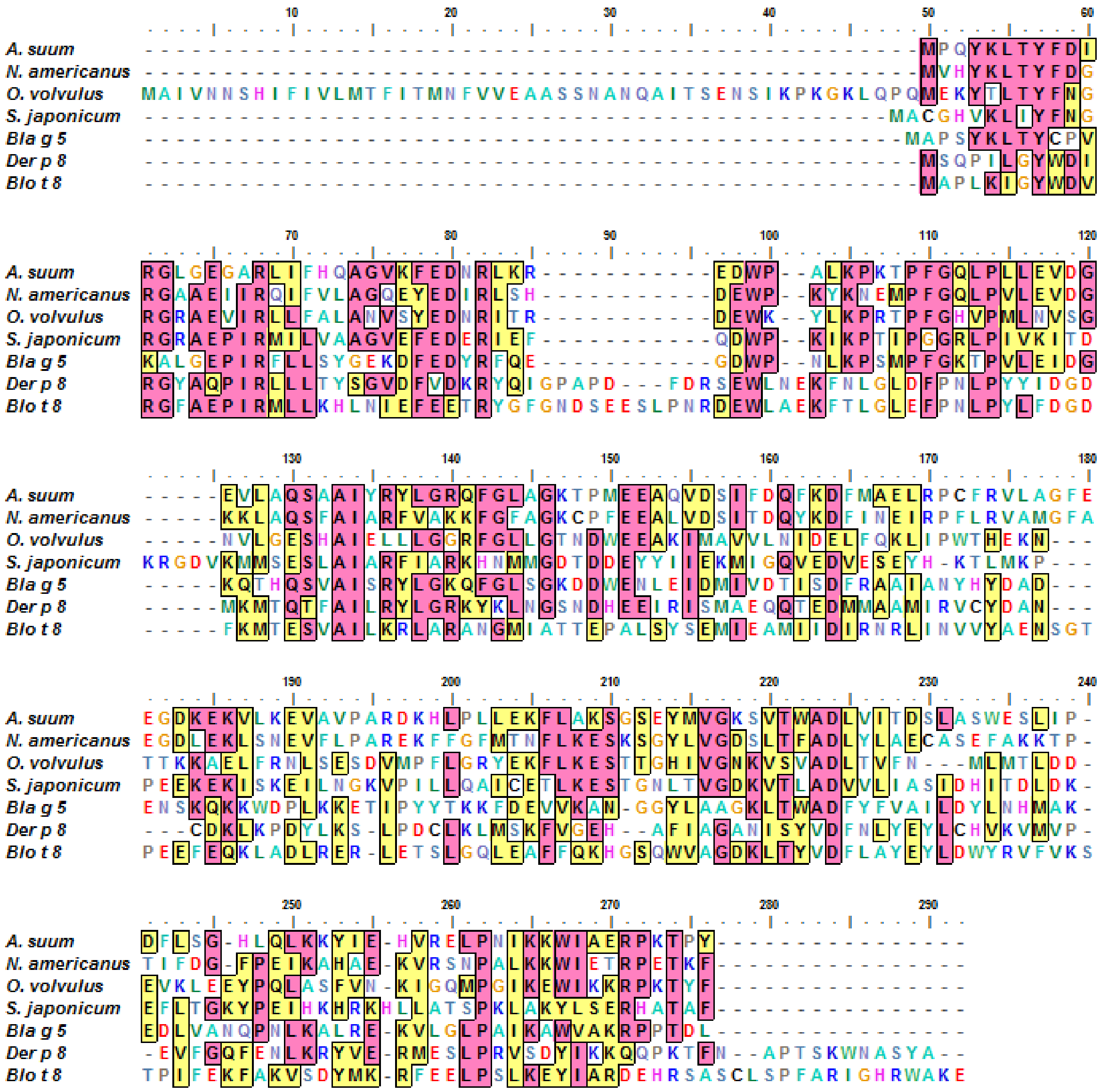

Amino acid composition of the Patches of conserved and surface-exposed residues between Ascaris GST1 and non-parasitic GSTs from mites (Der p 8, Blo t 8) and cockroach (Bla g 5)

Bla g 5 and Ascaris GST1

Bla g 5 and Ascaris GST1 have 50% of sequence similarity.

Patch #1:
P3, K6, E31, D32, E56, D58, G59, K60 →Bla g 5
P2, K5, E30, D31, E55, D57, G58, E59 → GSTA

Patch#2:
Q64, A67, R70, Y71, K74, Q75, G77, G80, D90, D94, D98 → Bla g 5
Q63, A66, R69, Y70, R73, Q74, F75, G76, D89, D93, D97 → GSTA

Patch#3:
P41, K44, P45, F49, K51 →Bla g 5
P40, K43, P44, F48, Q50 → GSTA

Patch#4:
P192, K195, A199, E200, P202 →GSTA
P190, K193, A197, K198, P201 →Bla g 5

Blo t 8 and Ascaris GST1

Blo t 8 and Ascaris GST1 have 43% of sequence similarity

Patch #1:
K200, E204, P206, K209, E210, I212, A213 → Blo t 8
E186, E190, P192, K195, K196, I198, A199 → GSTA

Patch #2:
D10, R33, N46, R47, D48, E49, A52 → Blo t 8
D10, R33, K35, R36, E37, D38, A41 → GSTA

Der p 8 and Ascaris GST1

Der p 8 and AscGST have 48% of sequence similarity

Patch #1:
D66, G67, D68, M69, M71, Q73, A76, R79, R83, K84 → Der p 8
D57, G58, E59, V60, L61, Q63, A66, R69, R73, Q74 → GSTA

Patch #2:
Q104, E106, D107, A111, R114 → Der p 8
Q94, K96, D97, A100, R103→ GSTA

Patch #3:
M171, P173, E174, K183 R184 → Der p 8
P172, D173, K182, K183, E186 → GSTA
