# Supplementary material for: Proteomic and Immunochemical Characterization of Glutathione Transferase as a New Allergen of the Nematode Ascaris lumbricoides
Source: PLoS One. 2013 Nov 4;8(11):e78353. doi: 10.1371/journal.pone.0078353 (PMC3817249; doi:10.1371/journal.pone.0078353)
Supplement: File S3 — Tryptic peptides coinciding with known GST sequences after LC-MS/MS of purified nGSTA. (DOCX) [file pone.0078353.s003.docx]

| **Mass** | **M/Z** | **Delta(ppm)** | **Modifications** | **Sequence** | **Species** | **Sequence ID.** |
| --- | --- | --- | --- | --- | --- | --- |
| 3306.5146 | 827.69 | -64.7502 | Carbamidomethyl C (25) | TPMEEAQVDSIFDQFKDFMAELRPCFR | *Ascaris suum* | GST1_ASCSU |
| 2586.3694 | 863.1643 | -38.7005 | None | TPFGQLPLLEVDGEVLAQSAAIYR | *Ascaris suum* | GST1_ASCSU |
| 2083.085 | 695.3744 | -7.032 | None | PQYKLTYFDIRGLGEGAR | *Ascaris suum* | GST1_ASCSU |
| 1914.0481 | 639.0357 | -18.6222 | None | LIFHQAGVKFEDNRLK | *Ascaris suum* | GST1_ASCSU |
| 1883.8616 | 942.9428 | -4.471 | None | TPMEEAQVDSIFDQFK | *Ascaris suum* | GST1_ASCSU |
| 1672.869 | 558.6055 | 45.3168 | None | LIFHQAGVKFEDNR | *Ascaris suum* | GST1_ASCSU |
| 1559.7849 | 520.9461 | -19.2518 | None | IFHQAGVKFEDNR | *Ascaris suum* | GST1_ASCSU |
| 1446.7008 | 724.3637 | -7.594 | None | FHQAGVKFEDNR | *Ascaris suum* | GST1_ASCSU |
| 1442.7557 | 722.3805 | 7.1918 | None | PQYKLTYFDIR | *Ascaris suum* | GST1_ASCSU |
| 1320.6565 | 661.3466 | -15.9904 | None | VLAGFEEGDKEK | *Ascaris suum* | GST1_ASCSU |
| 1259.666 | 630.8516 | -17.1522 | None | WIAERPKTPY | *Ascaris suum* | GST1_ASCSU |
| 1108.504 | 555.2596 | 0.4405 | None | AGFEEGDKEK | *Ascaris suum* | GST1_ASCSU |
| 1091.6343 | 546.8364 | -21.0224 | None | DKHLPLLEK | *Ascaris suum* | GST1_ASCSU |
| 1082.5765 | 542.3126 | -30.5568 | None | EDWPALKPK | *Ascaris suum* | GST1_ASCSU |
| 1080.6655 | 541.3388 | 3.2758 | None | VLKEVAVPAR | *Ascaris suum* | GST1_ASCSU |
| 1037.4669 | 519.751 | -18.7079 | None | GFEEGDKEK | *Ascaris suum* | GST1_ASCSU |
| 1011.5866 | 506.7986 | 4.9476 | None | LIFHQAGVK | *Ascaris suum* | GST1_ASCSU |
| 983.5399 | 984.5616 | -14.0867 | None | EVAVPARDK | *Ascaris suum* | GST1_ASCSU |
| 956.4271 | 957.4461 | -11.6782 | None | SGSEYMVGK | *Ascaris suum* | GST1_ASCSU |
| 926.4861 | 927.4324 | 66.4095 | None | LTYFDIR | *Ascaris suum* | GST1_ASCSU |
| 785.4185 | 786.4459 | -24.9444 | None | FHQAGVK | *Ascaris suum* | GST1_ASCSU |
| 740.4181 | 741.2903 | 183.2005 | None | EVAVPAR | *Ascaris suum* | GST1_ASCSU |
| 719.3972 | 720.4015 | 4.9209 | None | QFGLAGK | *Ascaris suum* | GST1_ASCSU |
| 679.2927 | 680.3069 | -9.3444 | None | FEDNR | *Ascaris suum* | GST1_ASCSU |
| 652.4274 | 653.4406 | -8.2324 | None | PALKPK | *Ascaris suum* | GST1_ASCSU |
| 591.3402 | 592.3539 | -9.9085 | None | FGLAGK | *Ascaris suum* | GST1_ASCSU |
| 512.3079 | 513.3089 | 13.3436 | None | AVPAR | *Ascaris suum* | GST1_ASCSU |
| 444.2702 | 445.2854 | -16.6231 | None | GLAGK | *Ascaris suum* | GST1_ASCSU |
| 719.4333 | 720.4475 | -8.9079 | None | KFGLAGK | *C. elegans* | GST4_CAEEL |
| 591.3402 | 592.3539 | -9.9085 | None | FGLAGK | *C. elegans* | GST4_CAEEL |
| 584.3283 | 585.342 | -10.0274 | None | AEPIR | *C. elegans* | GST4_CAEEL |
| 444.2702 | 445.2854 | -16.6231 | None | GLAGK | *C. elegans* | GST4_CAEEL |
| 1216.6604 | 609.3306 | 12.1404 | None | GYKVTYFAIR | *Ascaris suum* | GST2_ASCSU |
| 868.4806 | 869.4928 | -5.06 | None | VTYFAIR | *Ascaris suum* | GST2_ASCSU |
| 925.5021 | 463.771 | -26.2467 | None | LTYFNLR | *Gallus gallus* | GST5_CHICK |

**Supplementary file S3.** Tryptic peptides coinciding with known GST sequences after LC-MS/MS of the purified nGSTA
