# Supplementary material for: Proteomic and Immunochemical Characterization of Glutathione Transferase as a New Allergen of the Nematode Ascaris lumbricoides
Source: PLoS One. 2013 Nov 4;8(11):e78353. doi: 10.1371/journal.pone.0078353 (PMC3817249; doi:10.1371/journal.pone.0078353)
Supplement: File S5 — Peptides identified in three spots containing nGSTA after 2D electrophoresis and LC-MS/MS analysis. (PDF) [file pone.0078353.s005.pdf]

Supplementary File S5-A. Peptide identifications on spot 29

Mass	M/Z	Delta(ppm)	Modifications	Sequence	Organism	Entry
1883.8612	942.9428	-4.6654	None	TPMEEAQVDSIFDQFK	*Ascaris suum*	GST1_ASCSU
1320.6561	441.2295	-6.7475	None	VLAGFEEGDKEK	*Ascaris suum*	GST1_ASCSU
1063.5186	532.7781	-20.6599	None	VLAGFEEGDK	*Ascaris suum*	GST1_ASCSU
1011.5865	506.8106	-18.8848	None	LIFHQAGVK	*Ascaris suum*	GST1_ASCSU
956.4273	479.2321	-22.2074	None	SGSEYMVGK	*Ascaris suum*	GST1_ASCSU
926.4861	464.2515	-1.3834	None	LTYFDIR	*Ascaris suum*	GST1_ASCSU
815.429	408.732	-23.727	None	YIEHVR	*Ascaris suum*	GST1_ASCSU
996.5855	499.3102	-19.3528	None	DILPVELAK	*Wuchereria bancrofti*	Q86LL8_WUCBA
868.4807	435.2536	-12.5093	None	VTYFAIR	*Ascaris suum*	GST2_ASCSU

Supplementary File S5-B. Peptide identifications on spot 13

Mass	M/Z	Delta(ppm)	Modifications	Sequence	Organism	Entry
1883.8612	942.9659	-29.1582	None	TPMEEAQVDSIFDQFK	*Ascaris suum*	GST1_ASCSU
1440.6641	481.2327	-7.3716	Carbamidomethyl C (9)	DFMAELRPCFR	*Ascaris suum*	GST1_ASCSU
1320.6561	441.2217	10.907	None	VLAGFEEGDKEK	*Ascaris suum*	GST1_ASCSU
1091.6339	364.8858	-0.1118	None	DKHLPLLEK	*Ascaris suum*	GST1_ASCSU
1082.576	542.3048	-16.5753	None	EDWPALKPK	*Ascaris suum*	GST1_ASCSU
1063.5186	532.7766	-17.9053	None	VLAGFEEGDK	*Ascaris suum*	GST1_ASCSU
1011.5865	506.8058	-9.352	None	LIFHQAGVK	*Ascaris suum*	GST1_ASCSU
926.4861	464.2283	48.6863	None	LTYFDIR	*Ascaris suum*	GST1_ASCSU
848.512	425.2708	-16.4722	None	HLPLLEK	*Ascaris suum*	GST1_ASCSU
840.5069	421.2677	-15.322	None	ELPNIKK	*Ascaris suum*	GST1_ASCSU
815.429	408.7241	-4.3413	None	YIEHVR	*Ascaris suum*	GST1_ASCSU
719.3966	360.7067	-1.612	None	QFGLAGK	*Ascaris suum*	GST1_ASCSU
868.4807	435.254	-13.4229	None	VTYFAIR	*Ascaris suum*	GST2_ASCSU
754.4337	378.2266	-5.0968	None	GLAEPIR	*Dirofilaria immitis*	GSTP_DIRIM

Supplementary File S5-C. Peptide identifications on spot 12

Mass	M/Z	Delta(ppm)	Modifications	Sequence	Organism	Entry
1883.8612	942.923	16.3941	None	TPMEEAQVDSIFDQFK	*Ascaris suum*	GST1_ASCSU
1320.6561	441.2123	32.2597	None	VLAGFEEGDKEK	*Ascaris suum*	GST1_ASCSU
1091.6339	364.8864	-1.6773	None	DKHLPLLEK	*Ascaris suum*	GST1_ASCSU
1082.576	542.3043	-15.6733	None	EDWPALKPK	*Ascaris suum*	GST1_ASCSU
1063.5186	532.7742	-13.3143	None	VLAGFEEGDK	*Ascaris suum*	GST1_ASCSU
1011.5865	506.8097	-17.0748	None	LIFHQAGVK	*Ascaris suum*	GST1_ASCSU
956.4273	479.2177	7.8494	None	SGSEYMVGK	*Ascaris suum*	GST1_ASCSU
926.4861	464.2668	-34.4531	None	LTYFDIR	*Ascaris suum*	GST1_ASCSU
848.512	425.2685	-11.0055	None	HLPLLEK	*Ascaris suum*	GST1_ASCSU
840.5069	421.2648	-8.4235	None	ELPNIKK	*Ascaris suum*	GST1_ASCSU
815.429	408.7302	-19.311	None	YIEHVR	*Ascaris suum*	GST1_ASCSU
740.418	741.4347	-11.9527	None	EVAVPAR	*Ascaris suum*	GST1_ASCSU
719.3966	720.4158	-15.7804	None	QFGLAGK	*Ascaris suum*	GST1_ASCSU
